# Explaining socioeconomic disparities and gaps in the use of antenatal care services in 36 countries in sub-Saharan Africa

**DOI:** 10.1093/heapol/czab036

**Published:** 2021-04-13

**Authors:** Amarech G Obse, John E Ataguba

**Affiliations:** Health Economics Unit, School of Public Health & Family Medicine, University of Cape Town, Anzio Road, Observatory, Cape Town 7925, South Africa; Health Economics Unit, School of Public Health & Family Medicine, University of Cape Town, Anzio Road, Observatory, Cape Town 7925, South Africa

**Keywords:** Antenatal care, decomposition, inequality, sub-Saharan Africa

## Abstract

Significant maternal and child deaths occur in sub-Saharan Africa (SSA) even with existing effective interventions. Antenatal care (ANC), for example, is an intervention that improves the health of pregnant women and their babies, but only 52% of pregnant women in SSA had the recommended minimum of four ANC visits between 2011 and 2016. While significant socioeconomic inequalities in ANC visits have been reported to the disadvantage of the poor, little is known about the depth of ANC coverage and associated inequalities. This paper introduces ‘deficits’ (i.e. the number of ANC visits that are needed to reach the recommended minimum of four ANC visits) and ‘surpluses’ (i.e. the number of ANC visits over and above the recommended minimum of four ANC visits) to assess socioeconomic inequalities in the indicator and depth of the ‘deficits’ and ‘surpluses’ in ANC visits. Using the latest available Demographic and Health Survey data for 36 SSA countries and concentration indices, the paper found that ‘deficits’ in ANC visits are more prevalent among poorer women compared to ‘surpluses’ that are concentrated among the rich. On average, women with ‘deficits’ in ANC visits require about two more ANC visits to reach the recommended four ANC visits, and women with ‘surpluses’ exceeded the recommended minimum by about two ANC visits. The factors that explain a substantial share of the socioeconomic inequalities in ANC ‘deficits’ and ‘surpluses’ in SSA include wealth, education and area of residency, which are essentially the social determinants of health inequalities. For policy response, it is suggested that education is a significant channel to affect the other social determinants of inequalities in ANC coverage reported in the paper. Thus, countries must prioritize quality education as addressing education, especially among women in SSA, will significantly reduce disparities in ANC service utilization and accelerate progress towards universal health coverage.

**Key messages**

- In sub-Saharan Africa (SSA), poorer women, compared to richer women, are more likely to report fewer than the recommended four antenatal care (ANC) visits, while richer women are more likely to report more than four ANC visits.
- Women in SSA with less than four ANC visits have an average of two ANC visits fewer than the recommended minimum of four ANC visits, while women with more than four ANC visits report about two ANC visits above the recommended minimum.
- Wealth, education and residential area contributed substantially to the socioeconomic inequalities in ANC visits in SSA countries. These are critical social determinants of health inequalities.
- SSA countries must prioritize quality education as education is the key determinant that affects the other factors explaining socioeconomic inequalities in ANC visits in SSA.

## Introduction

Globally, pregnancy and childbirth-related causes were implicated in 810 daily maternal deaths in 2017, with two-thirds of the deaths occurring in sub-Saharan Africa (SSA) (World Health Organization, 2019b). SSA accounts for the highest share of the 2.5 million neonatal deaths (in 2018) and 2.6 million stillbirths (in 2015) recorded worldwide (Lawn *et al*., 2016; World Health Organization, 2019a). These mortalities occur amidst affordable and effective life-saving interventions (Campbell and Graham, 2006; Lawn *et al*., 2016). For example, among other things, significant reductions in maternal and perinatal morbidity and mortality are achievable through quality antenatal care (ANC) (World Health Organization, 2016). ANC provides a platform for risk diagnosis, prevention and treatment of pregnancy-related complications or concurrent diseases (Carroli *et al*., 2001). The health education and promotion components of ANC services serve as sources of knowledge and information for pregnant women to ensure positive pregnancy experience and best health conditions for both mother and baby (World Health Organization, 2016).

Previously, the World Health Organization (WHO) recommended a minimum of four antenatal care visits (ANC4+) for pregnant women and adolescent girls that should be provided by a skilled healthcare professional (World Health Organization, 2007a). Recently, a minimum of eight ANC contacts is suggested as necessary for an uncomplicated pregnancy (World Health Organization, 2016). Based on the previous recommendation (ANC4+), only 62% of pregnant women received ANC4+ between 2011 and 2016 globally, while even fewer women from SSA (52%) and South Asia (46%) had the recommended number of ANC visits (UNICEF, 2019).

In addition to the overall low coverage of ANC, especially in SSA and South Asia, substantial inequality exists in ANC4+ within countries and to the disadvantage of women and girls with poor living standards (World Health Organization, 2015; Abekah-Nkrumah, 2019). A recent cross-country study in SSA documented significant pro-rich inequalities in ANC4+ (Abekah-Nkrumah, 2019). Significant pro-rich income-related inequalities in attaining ANC4+ were also reported in another cross-country study for Nepal, Bangladesh, Ethiopia and Zimbabwe (Goli *et al*., 2018), with mother’s education explaining about 18.20% of the observed inequality in the use of ANC in Zimbabwe (Makate and Makate, 2017). In countries such as Chad, Ethiopia, Ghana, Lesotho, the Philippines, Namibia, Nepal, Malawi and Senegal, there is promising evidence of narrowing socioeconomic inequality in attaining ANC4+ (Ambel *et al*., 2017; Molina *et al*., 2013; Mehata *et al*., 2017; Abekah-Nkrumah, 2019). However, inequalities in attaining ANC4+ have widened for some countries like Bangladesh, Benin, Burkina Faso, Guinea, Niger, Togo, Zambia and Zimbabwe (Sanoussi, 2017; Abekah-Nkrumah, 2019; Rahman *et al*., 2017). The widening inequalities exist despite many governments’ actions at promoting reductions in inequalities—a global priority by the sustainable development goals (SDGs) (United Nations Development Programme, 2015).

Although a reduction in inequalities is necessary, it is important to understand the dynamics occurring among sub-populations within countries for policy targeting. For instance, in the case of Ethiopia, reductions in inequality in attaining the minimum of four ANC visits is primarily due to the middle-income population catching up with high-income population with the poorest still left behind (Ambel *et al*., 2017). Wealth, mother’s education and residential area are among strong predictors of ANC use (Rahman *et al*., 2017; Saad-Haddad *et al*., 2016; Gupta *et al*., 2017). These predictors also contributed most to inequality in less than four ANC visits. For instance, the largest contributors to inequalities in ANC coverage for Nepal were poor economic status (44%) and mother’s illiteracy (26%) compared to partner’s illiteracy (32%) and rural residence (25%) for Bangladesh (Goli *et al*., 2018). A recent study in Nigeria assessed socioeconomic inequalities in the intensity of ANC visits (i.e. the counts or number of ANC visits) (Nwosu and Ataguba, 2019) and found that in addition to the pro-rich distribution of attaining at least four ANC visits, poorer women have fewer ANC visits than richer women.

While such studies highlight inequalities in attaining ANC4+ or the number of ANC visits, there is a dearth of studies that examine how far off or closer women are to the recommended minimum number of ANC visits for uncomplicated pregnancies. The progressive realization of the goal of universal coverage, including access to ANC, must recognize the progress that countries make to increase coverage gradually (Ataguba, 2018). In fact, the Africa Agenda 2063 highlighted the plight of women and girls in Africa, stressing the need to achieve sound health and well-being (African Union, 2015). Closing existing gaps in health service coverage, including ANC, can help achieve good health and well-being for girls and women in Africa. While the traditional ANC4+ coverage is one of the tracer indicators of health service coverage for assessing progress towards UHC (i.e. an aspect of the SDGs), it does not capture how much less ANC services women are using or how many additional visits are required to attain the recommended minimum (Ataguba, 2018). Assessing the gaps in coverage provides a more holistic picture of ANC coverage relevant to assess the attainment of UHC. Thus, this paper suggests the need to go beyond the dichotomous split between attaining at least four ANC visits or not, to understanding what is called the depth (i.e. how far off women are from the recommended minimum) and assessing inequalities in the depth of coverage. Recently, Ataguba (2018) proposed a new index of ANC coverage that accounts for the depth of coverage. It is noted that two countries may have the same fraction of women attaining at least four ANC visits but with differences in the Ataguba’s (2018) index, reflecting differences in the depth (or quality) of ANC coverage between the countries.

This paper, therefore, assesses socioeconomic-related inequalities in the indicator of attaining at least four ANC visits and the depth of ANC visits in SSA countries where data are available. Socioeconomic inequalities in ANC utilization are also decomposed to explain significant factors that contribute to the observed inequalities.

## Methods

### Data and variables

Data come from the most recent Demographic and Health Survey (DHS) available for 36 SSA countries as of 20 April 2020 (see Table 1 for the years) (ICF, 2008–2018). The DHS is a representative survey conducted using very similar methodologies in all the countries to make them comparable. The survey is representative at the national, residence (urban–rural) and regional (departments, states) levels. DHS data are widely used for assessing maternal and child health issues in many developing countries, including SSA. The DHS uses a stratified two-stage cluster sampling design. At the first stage, enumeration areas (EAs) are sampled based on the country’s latest population and housing census. In the second stage, households are selected from each sampled EAs (Croft *et al*., 2018).

**Table 1.** ANC service coverage statistics, sub-Saharan Africa, various years

	Headcount (%)		Mean positive	
	Deficit	Surplus	Deficit	Surplus
Angola (2015/16)	42.37	44.63	2.75	2.29
Burkina Faso (2010)	65.37	5.54	1.59	1.58
Burundi (2018)	47.36	37.19	1.29	1.39
Cameroon (2011)	37.05	46.06	2.44	2.46
Chad (2014/15)	71.46	14.91	2.81	1.65
Comoros (2012)	41.10	46.36	1.95	2.96
Côte d’Ivoire (2011/12)	56.60	26.87	2.06	2.12
Democratic Republic of Congo (2013/14)	54.65	25.59	1.97	2.02
Eswatini (2006/07)	18.58	62.79	1.73	2.60
Ethiopia (2016)	63.74	20.42	2.89	2.10
Gabon (2012)	30.81	52.53	1.89	2.66
Ghana (2014)	13.51	75.75	2.02	3.49
Guinea (2018)	64.31	18.50	2.34	2.61
Kenya (2014)	45.68	30.04	1.66	2.01
Lesotho (2014)	25.23	53.84	1.86	2.68
Liberia (2013)	23.87	63.08	1.83	3.25
Madagascar (2008/09)	49.99	25.90	1.91	1.81
Malawi (2015/16)	49.00	21.33	1.40	1.77
Mali (2018)	56.90	24.26	2.63	2.38
Mozambique (2011)	44.84	31.81	1.98	1.62
Namibia (2013)	19.83	66.44	1.98	3.84
Niger (2012)	66.79	12.61	2.08	1.32
Nigeria (2018)	42.66	44.68	3.37	5.12
Republic of Benin (2011/12)	40.59	40.83	2.26	2.73
Republic of Congo (2011/12)	26.19	57.89	2.21	2.05
Rwanda (2014/15)	55.94	1.75	1.36	1.47
São Tomé and Principe (2008/09)	22.25	62.12	1.86	2.89
Senegal (2017)	45.52	11.57	1.65	1.86
Sierra Leone (2013)	12.96	74.94	1.79	5.05
South Africa (2016)	19.41	67.68	2.21	2.89
Tanzania (2015/16)	50.33	23.44	1.49	1.63
The Gambia (2013)	21.74	58.56	1.47	2.00
Togo (2013/14)	44.51	28.95	1.91	1.81
Uganda (2016)	39.89	20.15	1.46	1.88
Zambia (2016)	35.11	32.54	1.37	1.55
Zimbabwe (2015)	23.57	59.82	2.08	2.71

Deficit is an indicator that a woman had less than the recommended minimum of four visits; surplus is an indicator that a woman had more than the recommended minimum of four ANC visits.

Eligible women for this study were those who reported ANC visits for their most recent childbirth. The explanatory variables used in the decomposition are determined based on literature (Goli *et al*., 2017; Ambel *et al*., 2017; Babalola, 2014; Mustafa and Mukhtar, 2015; Saad-Haddad *et al*., 2016; Yaya *et al*., 2018) and availability in the DHS for all countries. These variables include age, the region of residence, urban/rural location, education, wealth, marital status, economic activity, decisions on health and major household purchases and birth order of the child. Household wealth is measured using a wealth index. Several household asset variables, including the ownership of consumer items, source of drinking water, sanitation facilities and type of flooring material, were used to construct the wealth index (ICF, 2020). Data are analysed using Stata 15 (StataCorp, 2017).

### Measures of ‘deficit’ and ‘surplus’ in ANC visits

The assessment of the depth (i.e. how far off women are from the recommended minimum) involved computing ‘deficits’ and ‘surpluses’ in ANC visits. In this paper, a ‘deficit’ in ANC visit is computed for women who have attained less than the recommended minimum of four ANC visits. For these women, the ‘deficit’ in ANC visits is the number of ANC visits required to meet the minimum of four visits. Analogous to the Foster–Greer–Thorbecke (FGT) metrics (Foster *et al*., 1984), let us define the ‘deficit’ in ANC visits for the $i$th woman (${D_i}$) as:

(1) \begin{equation*}{D_i} = \begin{cases} {{{\left( {4 - AN{C_i}} \right)}^\alpha }} & {{\rm{if\ }}AN{C_i} \lt 4}\\ {0,} & {{\rm{otherwise }}} \end{cases} \end{equation*}

where $AN{C_i}$ is the $i$th woman’s actual number of ANC visits, and $\alpha $ is a parameter that specifies the measure of interest. If $\alpha = 0$, we obtain an indicator for deficits (here, ${D_i}$ equals 1 for a woman with less than four ANC visits, and 0 otherwise), and $\alpha = 1$ is for the depth of the ‘deficit’ (i.e. the total number of ANC visits needed to attain the recommended minimum of four visits).

Similarly, the ‘surplus’ in ANC visits for the $i$th woman (${S_i}$) is expressed as:

(2) \begin{equation*}{S_i} = \begin{cases} {{{(AN{C_i} - 4)}^\alpha }} & { {\rm{if}}\ AN{C_i} \gt 4}\\ \cr {0,} & { {\rm{otherwise}} } \end{cases} \end{equation*}

Here, if $\alpha = 0$, we obtain an indicator for ‘surplus’ (${S_i}$ equals 1 for a woman with more than four ANC visits, and 0 otherwise), and $\alpha = 1$ is for the actual ‘surplus’ or depth of the ‘surplus’ (i.e. the total number of ANC visits over and above the recommended minimum of four visits).

## Analytical methods

### Assessing socioeconomic inequalities

Socioeconomic inequalities in the headcount (i.e. when $\alpha = 0$ in Equations (1) and (2)) and the depth (i.e. when $\alpha = 1$ in Equations (1) and (2)) for the ‘deficits’ and ‘surpluses’ were assessed using the concentration index (CI) (O’Donnell *et al*., 2008; Kakwani, 1980). Specifically, the FGT-CIs (i.e. CIs for the FGT metrics) laid out in Bilger *et al*. (2017) were computed. Just like the original CI, the values of the FGT-CI vary between −1 and +1. A positive FGT-CI means that the variable of interest, e.g. the indicator for ‘deficits’ in ANC, is concentrated among the rich (i.e. pro-rich) while a negative index signifies the opposite (i.e. pro-poor).

### Decomposing socioeconomic inequalities

The FGT-CIs were decomposed to explain factors that underlie the socioeconomic inequalities in the ‘deficits’ or ‘surpluses’ in ANC visits.

Let us denote the relationship between any of the measures of ANC ($H$) obtained in Equations (1) and (2) and relevant socioeconomic and demographic factors ($z$) as:

(3) \begin{equation*}{H_i} = \alpha + \mathop \sum \limits_k {\beta _k}{z_{ki}} + {\varepsilon _i}\end{equation*}

where $\alpha $ and $\beta $ are parameters, and $\varepsilon $ is the error term.

The FGT-CI can be written as:

(4) \begin{equation*}FGT\text{-}C{I_H} = \sum\limits_{k = 1}^k {\left( {{{{\beta _k}{{\bar z}_k}} \over {{\mu _H}}}} \right)C{I_k} + \left( {{{G{I_\varepsilon }} \over {{\mu _H}}}} \right)} \end{equation*}

where ${\mu _H}$ is the mean of $H$, obtained from Equation (1) or (2), $\left( {{{{\beta _k}{{\bar z}_k}} \over {{\mu _H}}}} \right)$ is the elasticity or responsiveness of $H$ to marginal changes in the *k*-th explanatory variable, while $C{I_k}$ is the CI for the *k*-th explanatory variable. $G{I_\varepsilon }$ denotes the generalized CI for the error term. $\left( {{{{\beta _k}{{\bar z}_k}} \over {{\mu _H}}}} \right)C{I_k}$ represents the contribution of the *k*-th explanatory variable to the socioeconomic inequality in the headcount or depth measures obtained in Equation (1) or (2). The last term, $\left( {{{G{I_\varepsilon }} \over {{\mu _H}}}} \right)$, represents the unexplained/residual component.

**Figure A1.**
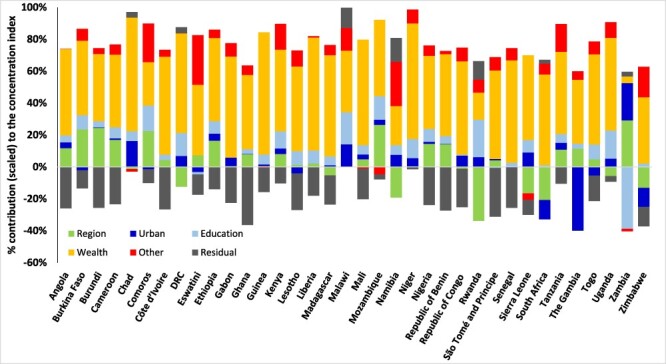
Decomposing the socioeconomic inequality in the intensity of the “surplus” in ANC visits, sub-Saharan Africa, various years.

As laid out in Bilger *et al*. (2017), the decomposition used a two-part model. The first part is for the headcounts (i.e. when $\alpha = 0$ in Equations (1) and (2)) with Equation (3) modelled using a non-linear (probit) model (van Doorslaer and Koolman, 2004). The second part is for the depths (i.e. when α=1 in Equations (1) and (2), corresponding to the deficits and surpluses, respectively) with Equation (3) modelled using a generalized linear model (GLM) that allows for flexibility in specifying different models, including count data found in this paper (Nelder and Wedderburn, 1972). The standard ‘linktest’ command in Stata (Pregibon, 1980) was used for diagnostics to choose the appropriate GLM model. While the Poisson and the negative binomial GLM family may be ideal for the depths in the ‘deficits’ and ‘surpluses’, respectively, the overall results of the decomposition (i.e. the contributions of the variables to the depth in ‘deficits’ and ‘surpluses’ inequalities) were not sensitive to the GLM specification. For brevity, the decomposition results for the depth in the ‘surpluses’ of ANC visits are presented in Figure A1 in Appendix 1. Moreover, we consider these results to be of less policy relevance as the progressive realization of ANC coverage seeks to focus on increasing the ANC coverage for women with less than the recommended minimum number of ANC visits (Ataguba, 2018). Also, the standard errors for the decomposed components in Equation (4) are obtained using bootstrap methods (Efron and Tibshirani, 1986) with 250 replications.

## Results

### ANC coverage in SSA

Data from the 36 countries indicate that between 13% and 71% of women aged 15–49 years with a live birth within a given period had less than the recommended minimum of four ANC visits in SSA (Table 1). The countries with more than 65% of women reporting less than four ANC visits (Chad, Niger, Burkina Faso and Guinea) and <15% of women reporting less than four ANC visits (Sierra Leone and Ghana) are in West Africa (Table 1). Similarly, between 2% and 76% of women had more than the recommended minimum of four ANC visits. Ghana, Sierra Leone, South Africa and Namibia had more than 65% of women exceeding four ANC visits, while Rwanda, Burkina Faso, Senegal, Niger and Chad had <15% of women exceeding four ANC visits (Table 1). On average, women in these SSA countries had about two ANC visits less (i.e. ‘deficit’) or more (i.e. ‘surplus’) than the recommended minimum of four visits.

### Socioeconomic inequality in ANC visits in SSA

**Figure 1.**
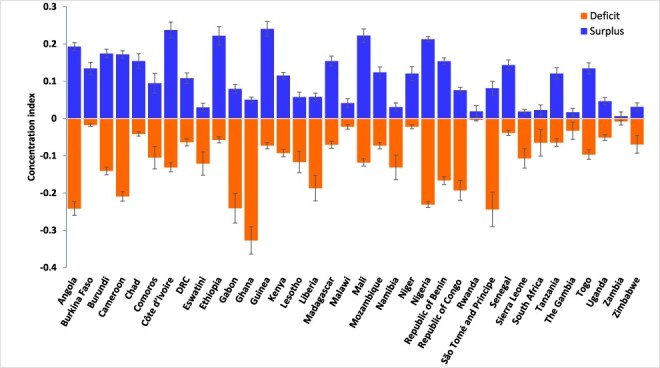
Socioeconomic inequality in ‘deficit’ or ‘surplus’ in ANC visits, sub-Saharan Africa, various years.

The CIs for indicators of ‘deficits’ and ‘surpluses’ in ANC visits (i.e. when $\alpha = 0$) were negative and positive, respectively, and are statistically significant at conventional levels for all the 36 countries (Figure 1). These results for the headcount show that poorer (richer) women are more likely to report ‘deficits’ (‘surpluses’) in ANC visits compared to richer (poorer) women. Figure 1 shows that the magnitude of the socioeconomic inequalities is different between countries. The CI for the indicator of ‘surpluses’ in ANC visits was >0.2 in Guinea, Côte d’Ivoire, Ethiopia, Mali and Nigeria but <0.02 in Zambia, Sierra Leone, Rwanda and the Gambia. Socioeconomic inequalities in the indicator of ‘deficits’ in ANC visits in Figure 1 are most pronounced in Ghana (CI = −0.33), São Tomé and Principe (−0.24), Angola (−0.24), Nigeria (−0.23) and Cameroon (−0.21).

DRC = the Democratic Republic of Congo; deficit is an indicator that a woman had less than the recommended minimum of four visits; surplus is an indicator that a woman had more than the recommended minimum of four ANC visits; error bars represent the 95% confidence interval.

**Figure 2.**
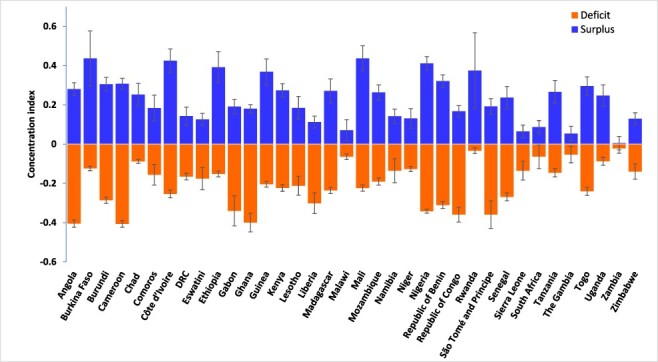
Socioeconomic inequality in the intensity of the ‘deficits’ or ‘surpluses’ in the ANC visits, sub-Saharan Africa, various years.

Similarly, the CIs for the depth of the ‘deficits’ or ‘surpluses’ in the ANC visits (i.e. when $\alpha = 1$) shown in Figure 2 are similar in pattern to those reported in Figure 1. The statistically significant positive CIs for the depth of ‘surpluses’ in ANC visits indicate that women from wealthier socioeconomic backgrounds do not only frequently exceed the recommended minimum of four ANC visits, but they tend to utilize more ANC services than their less wealthy counterparts. Also, poorer women tend to have fewer ANC visits than the recommended minimum, as seen in the statistically significant negative CIs for the depth of the ‘deficits’ in ANC visits in Figure 2. Socioeconomic inequalities in the depth of ‘surplus’ ANC visits are highest in Burkina Faso (CI = 0.44), Mali (0.44), Côte d’Ivoire (0.43) and Nigeria (0.41) but lowest in Zambia (<0.01), the Gambia (0.05) and Malawi (0.07). More pronounced inequalities in the depth of the ‘deficits’ in ANC visits are reported for Cameroon (−0.41), Ghana (−0.40) and Angola (−0.40) compared to Zambia (−0.02), Rwanda (−0.03) and the Gambia (−0.05) where the socioeconomic inequalities in the depth of the ‘deficits’ in ANC visits were less pronounced.

DRC = the Democratic Republic of Congo; intensity of deficits measures the number of ANC visits that a woman needs to reach the minimum of four ANC visits; the intensity of surplus is the number of ANC visits that a woman made over and above the recommended minimum of four ANC visits; error bars represent the 95% confidence interval.

### Explaining the socioeconomic inequality in ANC visits in SSA

**Figure 3.**
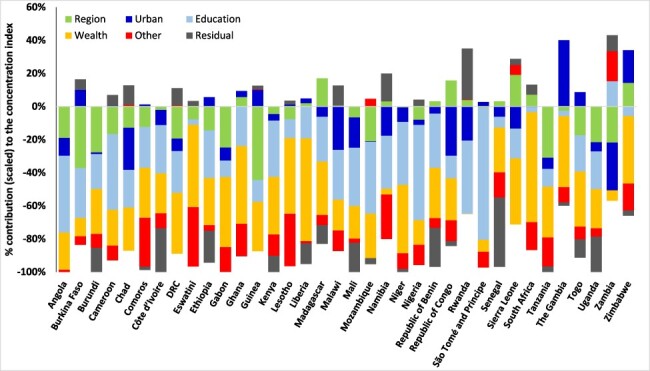
Decomposing the socioeconomic inequality in the indicator of the ‘deficit’ in ANC visits, sub-Saharan Africa, various years.

**Figure 4.**
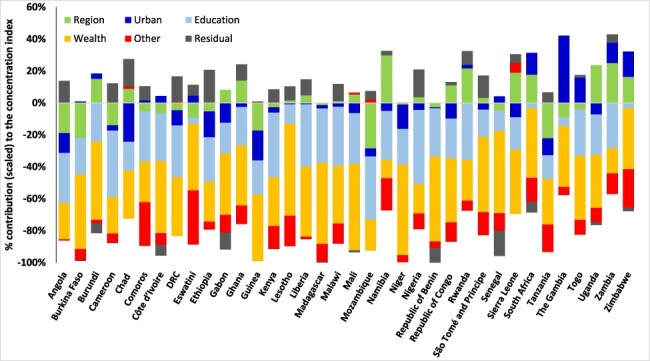
Decomposing the socioeconomic inequality in the intensity of the ‘deficit’ in ANC visits, sub-Saharan Africa, various years.

**Figure 5.**
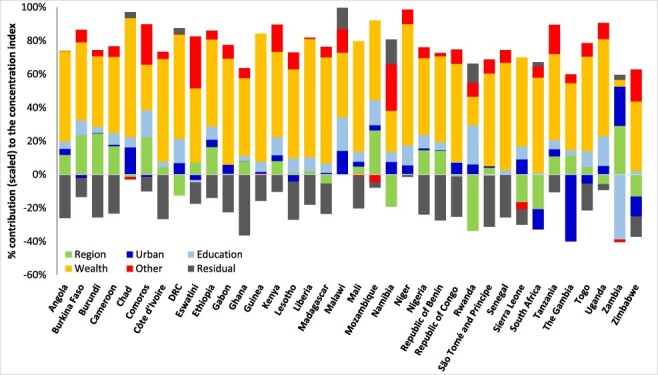
Decomposing the socioeconomic inequality in the indicator of the ‘surplus’ in ANC visits, sub-Saharan Africa, various years.

In all the 36 SSA countries, the relative contributions of wealth, education and the subnational region of residence, including urban/rural residence substantially, explain the bulk of the socioeconomic inequalities in the ‘deficits’ and ‘surpluses’ in ANC visits (Figures 3–5). Other factors included in the decomposition, such as the age of the woman, employment status, empowerment and the birth order of the children, explain very little of the socioeconomic inequalities in the indicator of ‘deficits’ or ‘surpluses’ in ANC visits in the countries. Education and wealth combined account for over 50% of the socioeconomic inequalities in ANC coverage reported in Figures 3–5. The residuals are substantial for a few countries because this paper used similar factors in the decomposition for all countries. Importantly, and as expected, socioeconomic inequalities in the indicator of ‘surplus’ ANC visits are explained primarily by the wealth level of women (Figure 5). As shown in Figure 5, wealth accounted for more than 70% of the pro-rich CIs in most countries.

DRC = the Democratic Republic of Congo; Other includes age, empowerment, cohabitation, planned pregnancy, employment status and birth order.

DRC = the Democratic Republic of Congo; other includes age, empowerment, cohabitation, planned pregnancy, employment status and birth order.

DRC = the Democratic Republic of Congo; other includes age, empowerment, cohabitation, planned pregnancy, employment status and birth order.

## Discussion

This paper demonstrates significant socioeconomic inequalities in ANC service coverage in SSA, to the disadvantage of poorer women. As reported in this paper, wealthier women, in all of the SSA countries included in this paper, are more likely to have four or more ANC visits compared to poorer women who also report fewer than four ANC visits. As expected, poorer women have significant ‘deficits’ in ANC visits, while wealthier women have significant ‘surpluses’ in ANC visits. In the case of Ghana, for example, where wealth-related inequality in the use of less than four ANC has widened between 2003 and 2014 (Asamoah and Agardh, 2017), the proportions of women with at least four ANC visits remained high (UNICEF, 2019). However, the concentration of the indicator of ‘deficits’ in ANC visits among the poor reported in this paper is significantly high, which shows that the lack of adequate ANC visits is prevalent among the poorest of the poor in Ghana. In other countries like Chad, Niger, Burkina Faso, Guinea and Ethiopia where a significantly high proportion of women have less than four ANC visits (UNICEF, 2019), pro-poor inequalities in the ‘deficits’ in ANC visits are relatively less pronounced because the ‘deficits’ in ANC utilization are prevalent across all socioeconomic groups. It is important to note that the pattern of socioeconomic inequalities in ANC utilization reported in this paper is not different between richer and poorer countries because, irrespective of the income or education attainment levels for the countries, for instance, there is a consistent pro-poor inequality in the ‘deficits’ in ANC visits and a pro-rich inequality in the ‘surpluses’ in ANC visits.

This paper also showed that, on average, women with ‘deficits’ in ANC visits require about two more ANC visits to realize the WHO recommended minimum of four ANC visits, and women with ‘surpluses’ in ANC visits exceed the recommended minimum by about two visits. The existence of inequality in ANC service usage to the advantage of the wealthy may lead to disproportionately higher morbidity and mortality related to pregnancy and childbirth among poorer population groups. The decomposition analysis reveals the significance of socioeconomic factors in addressing disparities in ANC service coverage in SSA. Key socioeconomic factors identified include wealth, education and place of residence (urban/rural and subnational regions). In addition to some context-specific factors (Say and Raine, 2007), these are critical social determinants of health inequalities (World Health Organization, 2013; Ataguba *et al*., 2015) that are crucial for reducing the significant disparities in health between and within countries.

After births attended by skilled health personnel, ANC utilization is the second largest contributor to inequalities in reproductive, maternal, new-born and child health services in low- and middle-income countries (LMICs) (World Health Organization, 2015). In LMICs, a minimum of 25 percentage point difference exists in having at least four ANC visits, between the advantaged and disadvantaged sub-populations (World Health Organization, 2015). As shown in this paper, several factors help explain gaps in service coverage, at least in relation to the minimum required for a healthy pregnancy experience. These crucial factors such as wealth (or income), education and residential area, reported in this paper as significant contributors to inequalities in ANC service coverage, have been noted elsewhere (Makate and Makate, 2017; Goli *et al*., 2017; Nwosu and Ataguba, 2019; Ambel *et al*., 2017). Although these previous studies did not categorize ANC service coverage adopted in this paper, the findings reveal the crucial role of the social determinants of health in reducing significant inequalities in health service coverage. In this paper, in almost all the countries, education contributed a significant share to the concentration of the ‘deficits’ and ‘surpluses’ in the ANC visits among poorer and wealthier women, respectively. Thus, low education attainment levels, found to be prevalent among poorer women in this study, may exacerbate ANC coverage inequalities among the poor and contribute to the improved ANC coverage among the wealthy. The place of residence, including urban and regional location, also contributed significantly to the pro-poor inequalities in the ‘deficits’ in ANC visits in most countries, albeit to a lesser extent than the contributions of education and wealth (Figures 3 and 4). A similar finding was reported in Zimbabwe although using rural residency status to assess inequalities in less than three ANC visits (Goli *et al*., 2017) and Nigeria (Nwosu and Ataguba, 2019). Thus, reducing disparities in residence status between the rich and the poor will significantly reduce the pro-poor inequalities in the ‘deficits’ in ANC visits reported in SSA.

Generally, it is established that adequate ANC service usage can significantly improve maternal and child health and save lives (Moyer and Mustafa, 2013). ANC coverage (especially having at least four visits) significantly predicts facility-based delivery (Choe *et al*., 2015), which is positively associated with improving maternal and newborn health (World Health Organization, 2007b). As the findings in this paper show, in addition to expanding coverage for ANC services, SSA countries need to make concerted efforts, including through the use of policies, to reduce coverage gaps and accelerate the reduction of inequalities. Reducing ANC coverage gaps (i.e. the deficits) is essential for improving access to health services for all pregnant women (an aspect of SDG 3). Significant reductions in maternal health inequalities through the use of ANC services are directly relevant to three SDGs—goals 3 (good health and well-being), 5 (gender equality) and 10 (reduced inequalities) (United Nations Development Programme, 2015). To achieve SDG 10, all aspects of inequality reduction are essential, including reducing ANC ‘deficits’ between the poor and rich as reported in this paper.

This paper goes beyond using indicators of attaining at least four ANC visits (Ambel *et al*., 2017; Asamoah and Agardh, 2017; Goli *et al*., 2017; Makate and Makate, 2017; Molina *et al*., 2013; Abekah-Nkrumah, 2019) or the count of ANC visits (Nwosu and Ataguba, 2019) that are common in previous studies to also contribute to addressing the related SDGs. It introduces the concept of ‘deficits’ and ‘surpluses’ in ANC visits to understand factors that explain socioeconomic inequalities in not only the indicators of ‘deficits’ and ‘surpluses’ but actual (i.e. the depth) ‘deficits’ and ‘surpluses’. Interestingly, many of the significant factors reported in this paper are primarily about the social determinants of health inequalities. Although these factors are similar to those reported in previous studies, they shed light on the importance of understanding the depths of inadequate ANC services utilization, which is predominant among poorer women. The importance of the social determinants of health means that policies to address socioeconomic inequalities in ANC services in SSA must be inherently intersectoral (Nwosu and Ataguba, 2019; World Health Organization, 2013). There is a positive correlation between education and wealth, including the region of residence, with higher returns for females than males (Patrinos and Psacharopoulos, 2020). We argue that quality education, which requires multisectoral actions, is the primary transmission unit for affecting the other critical social determinants of health underlying the significant socioeconomic inequalities in ANC coverage in Africa. Although there have been improvements in a few countries in Africa, education levels remain low, with educational attainment levels lower among women than men in many countries (Graetz *et al*., 2018). Countries like Botswana, Rwanda and South Africa may have attained relative parity in educational levels between men and women (Graetz *et al*., 2018), but there is still scope for increasing average educational attainment levels and education performance on the continent that are lower than expected. Focusing on the education of girls and women reduces the incidence of early child marriage (Efevbera *et al*., 2019) and also contributes to significantly reducing inequalities in ANC service utilization. The positive returns on education (Patrinos and Psacharopoulos, 2020) imply that improved education outcomes will have significant long-term impacts on wealth and region of residence, the major social determinants of inequalities in ANC service utilization reported in this paper.

Girls’ and women’s education is affected by many factors, including macro development, policies, legislation, institutional, sociocultural, community and household level issues (Kane, 2004). The institutional capacity, governance system and culture of countries vary considerably, requiring countries to adopt context-specific strategies for expanding access to quality education (beyond just attendance) for girls and women. Apart from the significant focus on ensuring universal primary education (d’Aiglepierre and Wagner, 2013), country strategies should also address the learning needs of out-of-school adolescent girls, assist the completion of secondary school, assist school-to-work transitions and empower girls and women (Sperling and Winthrop, 2015). These strategies are aligned with Africa’s Agenda 2063 that aims to eliminate barriers to quality health and education for women and girls in Africa (African Union, 2015).

While improving the education and living conditions of women can only be achieved in the long term, in the short term, countries need to adopt strategies that mitigate differences in the use of ANC due to differences in educational attainment, place of residence and wealth. Among effective interventions that improve ANC attendance is home visits by community health workers, who identify pregnant women and provide education and referral for the use of ANC services (Esopo *et al*., 2020; Olaniran *et al*., 2019). Innovative advertisement of ANC and behavioural interventions that included birth plan before delivery (Esopo *et al*., 2020) are also among the interventions with a positive effect on the use of ANC.

This paper provides the baseline for tracking and explaining socioeconomic inequality in the ‘deficits’ and ‘surpluses’ in ANC utilization at the global SSA level. However, within countries, more nuanced results can be obtained to understand and inform policy on the critical factors that explain socioeconomic inequalities in ANC service coverage by including locally relevant factors subsumed in the residuals (Figures 3–5). Such analyses could further disaggregate the population by region, states or residential areas. Although this paper provides cross-country assessments, it is critical to exercise caution in cross-country comparisons as data come from different years and different country contexts (Ataguba, 2019). Ideally, the magnitude of the residuals reported in Figures 3–5 should be close to zero (Wagstaff *et al*., 2003), but this was not achieved for all countries as the estimation used the same variables across countries. Interestingly, the magnitude of the residuals (in Figures 3–5) in most of the countries is relatively smaller than the effect of the leading social determinants of health—education and wealth. Also, the same GLM family (e.g. the Poisson model for the depth in ‘deficits’) was used for all countries to ensure uniformity. However, applying different GLM specifications did not change our results qualitatively, as has been reported elsewhere (Hosseinpoor *et al*., 2006), mainly because the focus is on the contributions of the socioeconomic and demographic factors to inequality as seen in Equation (4) and not directly the coefficients in Equation (3).

## Conclusion

Socioeconomic inequalities in the use of ANC services are prevalent in SSA to the advantage of wealthier women. These inequalities are driven mainly by vital social determinants of health inequalities such as education and wealth, including the place of residence. For adequate policy response to meet the SDGs, including the Agenda 2063 goals in Africa, education is a significant channel to affect other social determinants of health. In recognizing the centrality of education, countries must prioritize quality education to reduce significant disparities in ANC service utilization to improve the health of women and the population for realizing the SDGs.

## Figures and Tables

DRC = the Democratic Republic of Congo; other includes age, empowerment, cohabitation, planned pregnancy, employment status and birth order.
